# Gastric Mucormycosis in a Patient With Uncontrolled Diabetes: A Rare Presentation

**DOI:** 10.7759/cureus.108888

**Published:** 2026-05-15

**Authors:** Lakshmana Prasanth Katragadda, N. Senthil, A.K Koushik, Pavithra V, Viswanathan Pandurangan

**Affiliations:** 1 General Medicine, Sri Ramachandra Institute of Higher Education and Research, Chennai, IND; 2 Internal Medicine, Sri Ramachandra Institute of Higher Education and Research, Chennai, IND; 3 Gastroenterology and Hepatology, Sri Ramachandra Institute of Higher Education and Research, Chennai, IND; 4 Pathology, Sri Ramachandra Institute of Higher Education and Research, Chennai, IND

**Keywords:** diabetes mellitus, diabetic ketoacidosis, emphysematous pyelonephritis, endoscopy, gastric mucormycosis, gastrointestinal mucormycosis, histopathology, liposomal amphotericin b, sepsis, septic shock

## Abstract

Mucormycosis is a rapidly progressive, invasive fungal infection that predominantly affects immunocompromised individuals. Although rhino-orbital-cerebral disease is the most common clinical presentation, gastrointestinal mucormycosis is rare and associated with higher mortality rates. We report the case of a 55-year-old woman with uncontrolled diabetes mellitus and chronic airway disease who presented with septic shock secondary to emphysematous pyelonephritis and diabetic ketoacidosis. She was treated with insulin infusion and intravenous meropenem for 14 days. Despite recovery from acute kidney injury, she continued to experience persistent nausea and poor oral intake. Upper gastrointestinal endoscopy demonstrated gastric ulceration, and histopathological examination confirmed gastric mucormycosis. Liposomal amphotericin B was initiated with subsequent improvement in patient appetite and resolution of nausea. This case highlights the importance of early endoscopic evaluation and tissue diagnosis in high-risk patients with persistent gastrointestinal symptoms during recovery from critical illness.

## Introduction

*Mucormycotina* is a subphylum of fungi that includes species previously classified under Zygomycetes. The medically important order Mucorales comprises genera such as *Rhizopus*, *Mucor*, and *Rhizomucor*, which are ubiquitous in soil and decaying organic matter [1]. These organisms cause mucormycosis, an aggressive angioinvasive fungal infection that predominantly affects immunocompromised individuals, particularly those with uncontrolled diabetes mellitus, hematologic malignancies, or post-transplant states [1]. Fungal spores infect and invade the blood vessels, causing thrombosis, tissue ischemia, and necrosis.

Clinical features of mucormycosis vary by site and include facial swelling with black eschar, cough, hemoptysis, abdominal pain, nausea, and necrotic skin lesions. Among the various clinical forms, rhino-orbital-cerebral mucormycosis is the most common, accounting for approximately 34% of cases, followed by cutaneous (22%) and pulmonary (20%) involvement [2]. Although these proportions are derived from large historical datasets, more recent studies continue to confirm a similar distribution pattern, with rhino-orbital disease predominating.

Gastrointestinal mucormycosis is relatively uncommon and presents with abdominal pain, nausea, vomiting, gastrointestinal bleeding, and perforation. It constitutes approximately 2%-8% of all cases but is associated with a particularly poor prognosis due to delayed diagnosis and nonspecific presentation [3,4]. Within the gastrointestinal tract, the intestine is most frequently involved (approximately 64%), followed by the stomach (around 33%), underscoring the rarity of gastric mucormycosis [5]. We present a rare case of gastric mucormycosis in a diabetic woman.

## Case presentation

A 55-year-old female patient with type 2 diabetes mellitus, chronic obstructive airway disease, and bipolar disorder presented with fever with chills and rigors and shortness of breath for two days. She was not compliant with her diabetic medications for the preceding three months. She was initially admitted to another hospital, diagnosed with septic shock. In view of a low Glasgow Coma Scale, the patient was started on inotropes, noradrenaline, vasopressin, and mechanical ventilation. She was later transferred to our tertiary care center for further management.

On admission, the patient was intubated and on inotropes. Systemic examination revealed bilateral coarse crepitations. Laboratory findings are summarized in Table 1.

**Table 1:** Laboratory investigations

Parameter	Patient Value	Reference Range
Hemoglobin	5.8 g/dL	12–15 g/dL
Total leukocyte count	21,240 /mm³	4,000–11,000 /mm³
Polymorphs	82.2%	45–70%
Platelets	2.93 lakhs/mm³	1.5–4.5 lakhs/mm³
HbA1C	12.0%	4-6%
Blood urea nitrogen	118 mg/dL	7–18 mg/dL
Serum creatinine	5.1 mg/dL	0.6–1.3 mg/dL
Sodium	155 mmol/L	134–144 mmol/L
Potassium	5.4 mmol/L	3.5–5 mmol/L
Chloride	122 mmol/L	96–108 mmol/L
Bicarbonate	14 mmol/L	22–28 mmol/L
Urine RBCs	52 /HPF	<5 /HPF
Pus cells	11.55 /HPF	0–4 /HPF
Yeast buds	Positive	Negative

**Figure 1:**
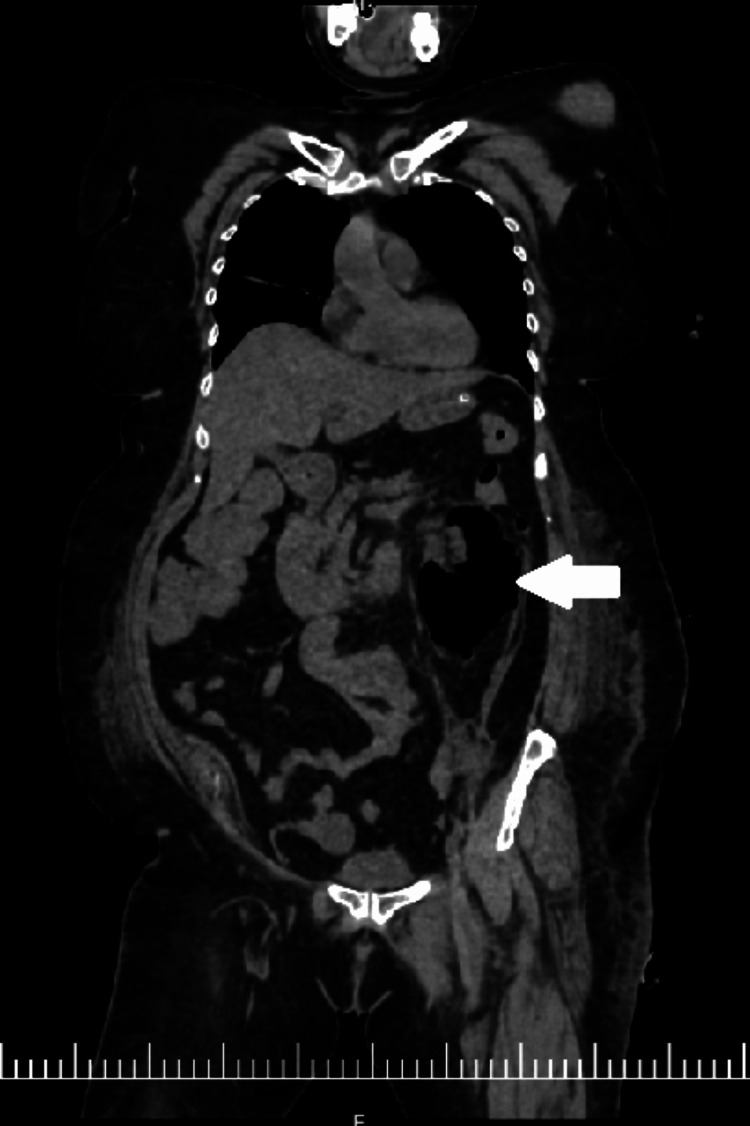
Computed tomography (CT) images of the kidneys, ureters, and bladder demonstrating a bulky left kidney with ill-defined collections and perinephric fat stranding, consistent with left emphysematous pyelonephritis.

Computed tomography of the kidneys, ureters, and bladder was suggestive of left emphysematous pyelonephritis (Figure 1).

**Figure 2:**
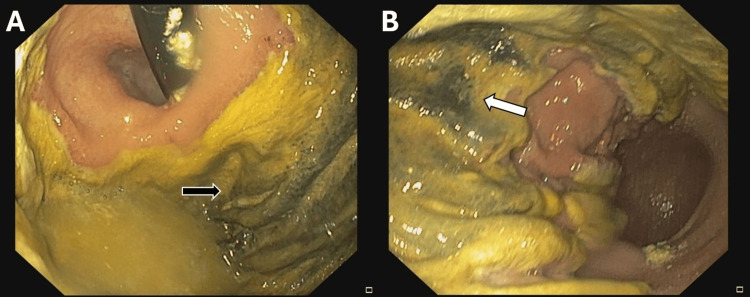
Upper gastrointestinal endoscopy. (A) Endoscopic view of the fundus of the stomach shows a pigmented ulcerated lesion (black arrow); (B) Endoscopic view of the body of the stomach shows a pigmented ulcerated lesion(white arrow).

The patient was initiated on human insulin infusion with hourly capillary blood glucose monitoring; ionotropes noradrenaline and vasopressin; and intravenous hydrocortisone, 50 mg every six hours. The patient underwent left double-J stenting and was managed with intravenous meropenem for 14 days; each dose of meropenem was given as an infusion for four hours. After clinical stabilization, inotropes were tapered, steroids were stopped, and she was extubated. Despite improved renal function (serum creatinine of 0.9 mg/dL), she had persistent vomiting and poor oral intake. An upper GI endoscopy revealed diffuse mucosal ulcerations with overlying slough predominantly along the greater curvature extending to the midbody of the stomach (Figures 2A, 2B).

**Figure 3:**
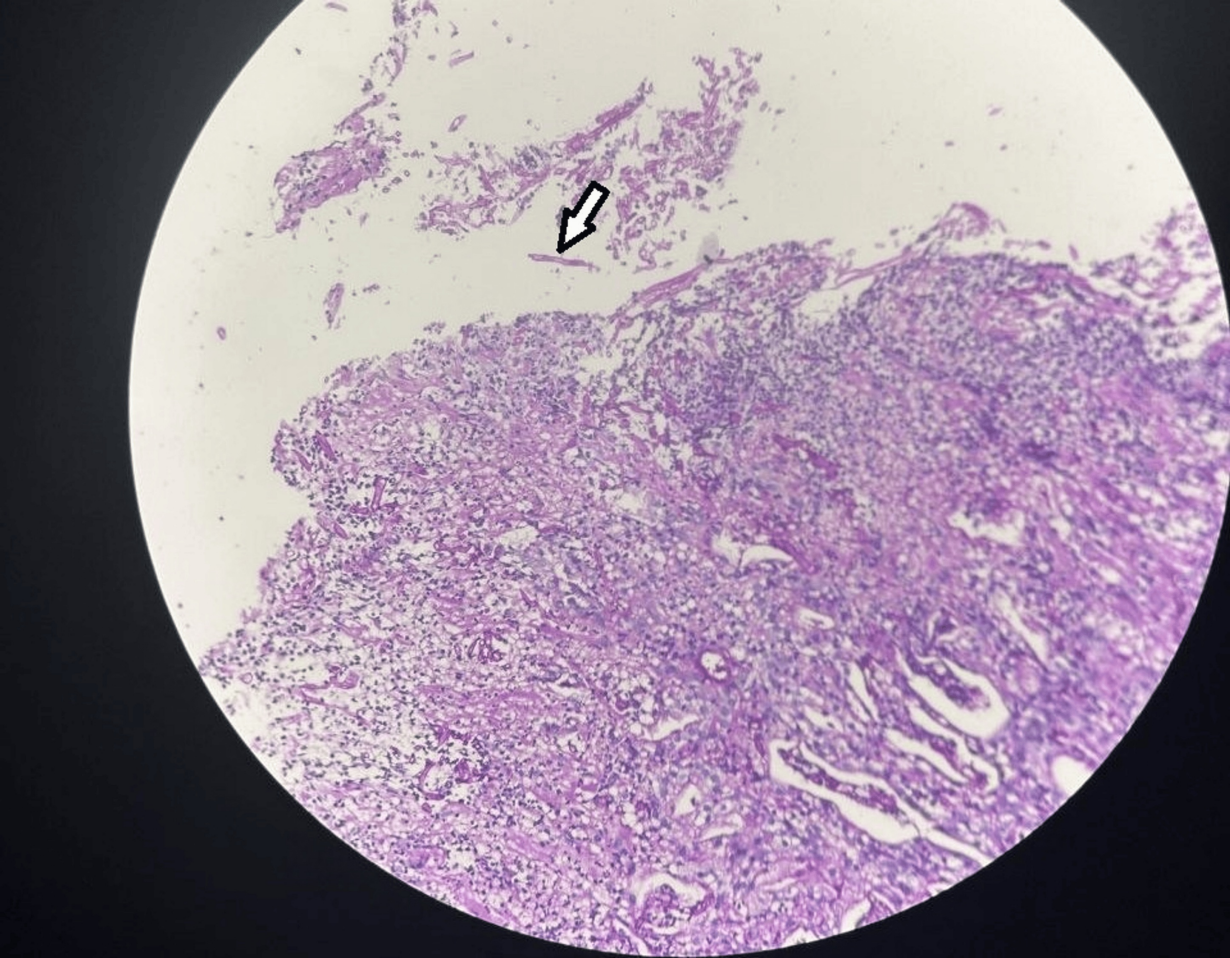
Periodic acid–Schiff stain shows the presence of broad aseptate hyphae resembling mucormycosis in the background of chronic gastritis.

Biopsies were obtained for histopathology (Figure 3).

Histopathology demonstrated broad, aseptate fungal hyphae consistent with *Mucor* species invading gastric glands, along with *Helicobacter pylori*. The patient was started on intravenous liposomal amphotericin B (5 mg/kg/day). Supportive management was continued, and glycemic control was optimized with insulin therapy.

**Figure 4:**
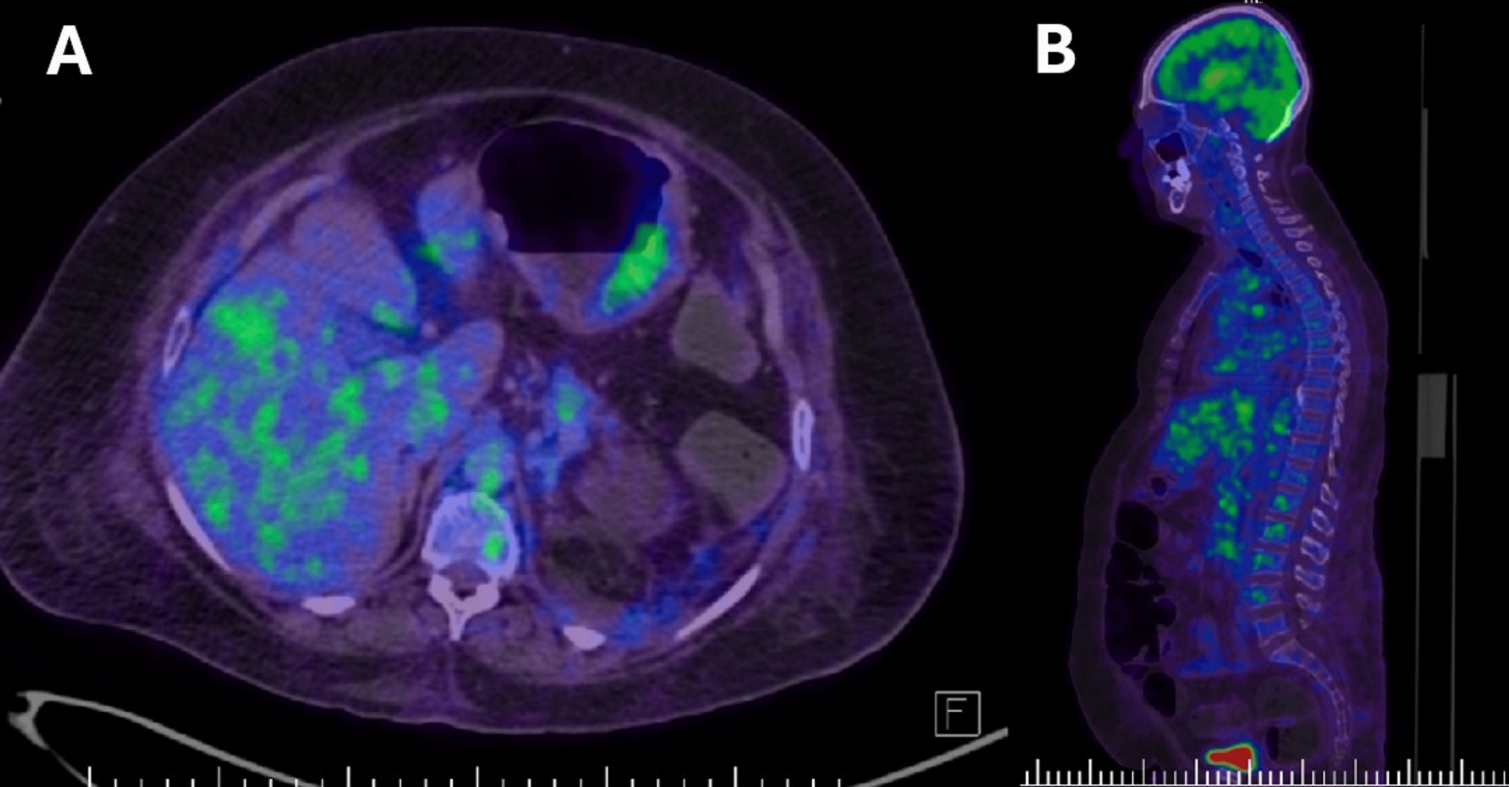
18F-FDG PET-CT images (A) Axial (transverse) PET-CT slice of the upper abdomen; (B) Sagittal PET-CT slice of the whole body. 18F-FDG: fluorine-18 fluorodeoxyglucose

A follow-up fluorine-18 fluorodeoxyglucose (18F-FDG) PET-CT showed mild FDG uptake in the gastric wall without any mass lesion or distant dissemination (Figure 4).

The patient showed gradual improvement with antifungal therapy and nutritional support. Surgical intervention was deferred due to clinical stabilization and lack of transmural necrosis. However, the patient was discharged against medical advice, citing personal reasons, and was lost to follow-up for further assessment and repeat endoscopy.

## Discussion

Gastric mucormycosis represents an infrequent yet highly invasive subtype of gastrointestinal mucormycosis, contributing to a small proportion of overall mucormycosis cases. Involvement of the gastrointestinal tract among all mucormycosis cases is 7%-13%, out of which the stomach is involved in 58% of the cases [6]. Despite its low incidence, it is associated with substantial mortality, largely due to delayed recognition and its rapid progression characterized by vascular invasion, thrombosis, and subsequent tissue necrosis [3,7].

Gastric mucormycosis is most commonly observed in patients with impaired immunity. Among the various risk factors, poorly controlled diabetes mellitus, particularly when complicated by ketoacidosis, remains the most significant. Additional contributing factors include prolonged corticosteroid therapy, hematological or solid organ malignancies, organ transplantation, renal dysfunction, extended intensive care unit stays, and exposure to broad-spectrum antimicrobial agents [8].

The clinical presentation is often nonspecific, which contributes to diagnostic delays. Patients may report abdominal discomfort, nausea, vomiting, fever, or evidence of gastrointestinal bleeding. As the disease advances, severe complications such as gastric wall necrosis, perforation, and life-threatening hemorrhage may develop, significantly worsening patient outcomes. Gastropleural fistula is one of the fatal and rare complications reported in a case [9]. Within the gastrointestinal tract, the stomach remains the most frequently involved organ [7].

Establishing a diagnosis can be challenging because the presenting features overlap with more common gastrointestinal conditions such as peptic ulcer disease or gastritis. Imaging studies typically lack specific diagnostic features. Definitive diagnosis relies on histopathological evaluation, which reveals characteristic broad, ribbon-like, aseptate hyphae with right-angle branching and evidence of angioinvasion [3]. Delayed diagnosis continues to be a major factor contributing to poor prognosis.

Effective management requires a combined approach. Early initiation of antifungal therapy, particularly with liposomal amphotericin B, is critical. In many cases, surgical intervention in the form of debridement or resection is necessary due to extensive tissue necrosis, and timely surgery has been shown to improve survival outcomes [3,10]. Concurrently, it is essential to address and reverse underlying predisposing factors wherever possible.

The overall prognosis of gastric mucormycosis remains unfavorable, with reported mortality rates ranging between 30% and 60% approximately depending on the timing of diagnosis and treatment as well as the extent of disease [9]. Factors associated with poorer outcomes include delayed initiation of therapy, disseminated infection, hemodynamic instability, and extensive gastric involvement.

In a report review of 20 cases of gastric mucormycosis, observed survivors were 50% (n=10), and mortality was 50% (n=10) [9].

This case underscores the importance of maintaining a high degree of clinical suspicion for mucormycosis in patients with relevant risk factors presenting with atypical gastrointestinal symptoms. Prompt endoscopic evaluation with tissue biopsy, early antifungal therapy, and timely surgical management are crucial steps that can significantly influence prognosis.

## Conclusions

Gastric mucormycosis is a rare but life-threatening infection that should be considered in high-risk patients, particularly those with uncontrolled diabetes and persistent gastrointestinal symptoms. Early suspicion, prompt endoscopic biopsy, rapid initiation of antifungal therapy, and correction of underlying risk factors are essential to improve outcomes. This case emphasizes the need for timely diagnosis and multidisciplinary management in preventing the high mortality associated with this condition.
